# Linguistically-Based Comparison of Different Approaches to Building Corpora for Text Simplification: A Case Study on Italian

**DOI:** 10.3389/fpsyg.2022.707630

**Published:** 2022-03-08

**Authors:** Dominique Brunato, Felice Dell'Orletta, Giulia Venturi

**Affiliations:** ItaliaNLP Lab, Institute for Computational Linguistics “A. Zampolli” (ILC-CNR), Pisa, Italy

**Keywords:** text simplification, aligned corpora, linguistic complexity, Italian language, corpus construction

## Abstract

In this paper, we present an overview of existing parallel corpora for Automatic Text Simplification (ATS) in different languages focusing on the approach adopted for their construction. We make the main distinction between manual and (semi)–automatic approaches in order to investigate in which respect complex and simple texts vary and whether and how the observed modifications may depend on the underlying approach. To this end, we perform a two-level comparison on Italian corpora, since this is the only language, with the exception of English, for which there are large parallel resources derived through the two approaches considered. The first level of comparison accounts for the main types of sentence transformations occurring in the simplification process, the second one examines the results of a linguistic profiling analysis based on Natural Language Processing techniques and carried out on the original and the simple version of the same texts. For both levels of analysis, we chose to focus our discussion mostly on sentence transformations and linguistic characteristics that pertain to the morpho-syntactic and syntactic structure of the sentence.

## 1. Introduction and Motivation

Automatic Text Simplification (ATS) is the Natural Language Processing (NLP) task aimed at reducing linguistic complexity of texts, especially at the lexical and syntactic levels, while preserving their original content (Bott and Saggion, 2014; Shardlow, 2014; Alva-Manchego et al., 2020a). It has long attracted the attention of different research communities that address the issue of generating a simplified version of an input text from two broad perspectives. The first perspective can be called “machine-oriented,” in that it conceives the task as a pre-processing step useful to improve the performance of other NLP tasks by providing an input that is easier to analyze for, e.g., syntactic parsing (Chandrasekar et al., 1996), Machine Translation (Štajner and Popović, 2016), Information Extraction (Klebanov et al., 2004; Niklaus et al., 2016), or Semantic Role Labeling (Vickrey and Koller, 2008) systems.

The second perspective is “human-oriented” and is concerned with the production of texts equally accessible for a wide variety of readers, also including less-skilled ones. In this respect, ATS is tightly intertwined with the Automatic Readability Assessment (ARA) task (Collins-Thompson, 2014), as they both share the primary objective of identifying and modeling properties of linguistic complexity within text according to cognitive and psycho-linguistic evidence on human sentence processing. However, while ARA allows discriminating between difficult-to-read and easy-to-read texts, ATS takes a step further which is to automatically convert the former into the latter. In this sense, it can be viewed as a sort of monolingual translation process. The two tasks have also in common the idea that linguistic complexity is a property highly related to the final reader. From this perspective, ATS studies have been devoted to define strategies to simplify texts for people with cognitive disabilities (Bott and Saggion, 2014), language impairments, e.g., aphasia (Carroll et al., 1998), dyslexia (Rello et al., 2013), or deafness (Inui et al., 2003), limited literacy skills (Aluísio et al., 2008) or a low proficiency level in a second language (Petersen and Ostendorf, 2007; Crossley et al., 2012). As most of our daily interaction with society, government, and other institutions require access to information conveyed by text, making content accessible also for this kind of people is ultimately a strategy to promote social inclusion. With this purpose in mind, numerous initiatives have been pursued in recent years by private and public organizations aimed at developing educational and assistive technologies for the benefit of human readers. A well-known one concerns the most popular free online encyclopedia, Wikipedia, which has been offering an easy–to–read version of its contents since 2003, although only limited to the English language^1^.

Another coarse distinction that has characterized the field of ATS is related to the methodological framework. Independently from the machine- or human-oriented purpose for which ATS is carried out, early computational approaches to the task were mostly based on hand-crafted rules targeting specific complex constructions informed by theoretical, cognitive, and computational linguistics literature. A special focus was put on the syntactic level with specific rules addressing the simplification of relative clauses, appositions, subordination, passive voice (Chandrasekar et al., 1996; Siddharthan, 2002, 2010). These methods can reach high precision and can potentially account for the maximum linguistic information, but they are extremely time-consuming and tend to cover only a few lexical and syntactic constructions. To overcome these drawbacks, much of current research is shifting toward data-driven techniques, most recently based on neural sequence-to-sequence models (Nisioi et al., 2017), which can automatically acquire from corpora the transformations occurring to sentences when they are manually simplified. The first fundamental requirement to allow the application of data-driven methods is the availability of large–scale monolingual parallel corpora, i.e., corpora containing the original and the simplified version of the same text possibly aligned at the sentence level. This is the main goal of the initiative launched by Wikipedia devoted to leveraging volunteers to create pages more easy-to-be read by everyone, both children and adults who are learning English. The efforts resulted in the first resource used for ATS purposes that include portions of English (EW) Wikipedia automatically aligned to Simple English Wikipedia (SEW) ones. It represented a benchmark because of its size and availability, mostly used by many ATS studies allowing both the development of machine learning algorithms and the evaluation of the quality of the automatic simplification results (Alva-Manchego et al., 2020b).

However, beyond size, there is a widespread consensus in the community that the quality of the simplified language resources is of fundamental importance and needs to be investigated in detail. This issue can be easily explained by drawing parallelism with the translation studies: in order to assess how good are simplified language resources, there should be a *native simplified–language speaker* who masters the target and the source language thus guaranteeing the quality of the translated texts (Siddharthan, 2014). But it is not the case since, as we introduced above, simplified texts should be differently conceived to reach a large number of target readers. This means that different varieties of simplified language exist with different characteristics that should be learned by ATS systems. In light of these considerations, the suitability of the Wikipedia-based resource for ATS applications has become quite debated (Bach et al., 2011; Woodsend and Lapata, 2011; Yasseri et al., 2012) and brought to the creation of new resources for the English language. To date, the most important one is the Newsela corpus (Xu et al., 2015), which contains original sentences extracted from newspaper articles and their simplified version at different readability levels by professional editors. Unlike parallel corpora derived from the original and simple English Wikipedia, the manually performed simplification guarantees very consistent alignments at the sentence level, as well as high quality of the linguistic transformations undergone by the original sentences (Xu et al., 2015; Scarton et al., 2018). As shown in the following section, during the last few years other resources have been built, but the Newsela corpus represents nowadays the most comprehensive benchmark for the English language since it includes the widest and qualitatively checked range of simplification operations.

The number of outcomes in ATS research, both in terms of large-scale corpora and available systems, mostly concerns a highly-resource language like English. The picture is different in other languages, for which a preliminary step in the development of ATS systems has been typically represented by the collection of monolingual parallel corpora either from scratch, asking experts (e.g., teacher, translators, speech therapists) to simplify texts for a specific target or aligning existing resources of original and simplified versions of the same texts. It is worth noticing that, with only a few exceptions (see Section 2 for details), these corpora are smaller than the ones available for English and this has made it hardly feasible to use them as training data for pure ATS systems based on machine learning methods. It is the reason why similar resources were primarily collected to be used as reference corpora to identify the most frequent simplification operations occurring in manually-simplified texts or to train rule-based systems covering limited sets of simplification phenomena, as in the case of, e.g., Italian (Barlacchi and Tonelli, 2013), Basque (Aranzabe et al., 2013), French (Brouwers et al., 2014), and German (Suter et al., 2016). Despite the low amount of data, useful methodological insights come from the Statistical Machine Translation (SMT) community. Monolingual SMT approaches were firstly tested for example for the Portuguese language (Specia, 2010), and more recently, neural MT architectures are used to develop neural text simplification systems for Italian (Aprosio et al., 2019), German (Sauberli et al., 2020), and Russian (Dmitrieva and Tiedemann, 2021). In these cases, the problem of data scarcity is alleviated with data augmentation techniques or methods to generate new synthetic data that are added to the original too small training corpora. However, this poses several issues related to the quality of the training data and, as a consequence, of the resulting automatically simplified sentences.

### 1.1. Our Contribution

In the scenario outlined so far, the main purpose of this study is to provide a deeper investigation on the effects that different approaches to ATS resources may have on the simplified corpora, with a special emphasis on languages other than English. Specifically, we focus on Italian since it is the only language, among the less-resourced ones, for which not only there are resources representative of the manual and the (semi)automatic approach, but they are also large enough to allow a significant comparison. Rather than proposing a method to assess the impact of the approaches used to build a resource on the performance of ATS systems, our investigation intends to assess whether and to what extent different approaches to the construction of ATS resources can affect the linguistic characteristics of simplified sentences to their original versions. In this sense, the purpose of the study is to contribute to the discussion on the quality of ATS resources by providing a fine-grained analysis of the linguistic phenomena characterizing parallel corpora available for the same language but built with different approaches. The investigation is twofold and it consists, on the one hand, in the study of the distribution of transformations (mainly syntactic structures) that the original sentences undergo when they are simplified; on the other hand, it is based on the results of a linguistic profiling analysis carried out with NLP-based techniques that allow comparing the original/simplified sentences by accounting for the distribution of a wide range of morpho-syntactic and syntactic characteristics automatically extracted from the linguistically annotated pairs of sentences. The first type of comparison requires the contribution of human experts who explicitly annotate the considered resource for a set of sentence transformations, while the second one is completely automatic.

The rest of the paper is organized as follows: Section 2 reports a survey of existing ATS corpora for different languages, drawing a main distinction according to the approach adopted in their construction, i.e., manual vs. (semi-)automatic. Section 3 describes the simplification operations that have been detected across these corpora to classify the major structural transformations involved in the process of sentence simplification. Our two-fold methodology devoted to comparing original and simplified sentences is described in Section 4, where we show how it can be used to study which kind of sentence transformations characterize a manually and an automatically derived corpus and whether the distribution of the linguistic characteristics of the corpus is correlated with the building approach.

## 2. Manual vs. (Semi-)Automatic Approach to Corpora Construction

In this section, we present an overview of the main ATS corpora existing for different languages. As shown in Table 1, we classified them into two main typologies: the ones manually built and the ones built adopting automatic (or semi-automatic) methods. For each corpus, we further provide the following details: the textual genre, the language, the target user, and the dimension.

**Table 1:** Monolingual parallel corpora of original/simplified sentences classified with respect to the type of approach adopted for their construction, the language, the textual genre, the target (GP, general purpose; CHI, children; LL, language learners; L2LL, L2 language learners; PLI, people with language impairments; PLL, people with low literacy level; NLP, NLP tasks; CS, crowd-sourcing), and the size of corpus.

**Manual approach**			
**Language**	**Textual genre**	**Target**	**Dimension**
ENG (Pellow and Eskenazi, 2014)	Everyday documents	GP	200 sentence pairs
ENG (Xu et al., 2015)	Newspapers	CHI	56,037 original sentences
ENG (Barzilay and Elhadad, 2003)	Encyclopedia Britannica	CHI	2,600 easy-to-read documents
ENG (Allen, 2009)	Classroom materials	LL	178,967 of simplified words
ENG (Petersen and Ostendorf, 2007)	Newspapers	LL	2,539 original sentences
ENG (Xu et al., 2016)	Wikipedia	CS	2,359 original sentences
ENG (Alva-Manchego et al., 2020a)	Wikipedia	CS	2,359 original sentences
Many (Orasan et al., 2013)	Miscellanea	PLI	320 original sentences
SPA (Bott and Saggion, 2014)	Newspapers	PLI	145 simplified sentences
SPA (Collados, 2013)	Newspapers	NLP	300 simplified sentences
FRE (Brouwers et al., 2014)	Narrative texts	L2LL	83 original sentences
FRE (Grabar and Cardon, 2018)	Encyclopedic, scientific, clinical texts	GP	4,596 sentence pairs
FRE (Gala et al., 2020)	L1 student materials	PLI	52,704 tokens
DAN (Klerke and Søgaard, 2012)	Newspapers	L2LL	3,701 document pairs
POR (Caseli et al., 2009)	Newspapers	PLL	2,116 original sentences
POR (Aluísio et al., 2008)	Popular science articles	PLL	882 original sentences
GER (Klaper et al., 2013)	Websites	PLI	7,755 original sentences
GER (Sauberli et al., 2020)	Newspapers	L2LL	3,616 sentence pairs
JPN Goto et al. (2015)	Newspapers	L2LL	2,885 sentence pairs
EUS Gonzalez-Dios et al. (2017)	Popular science articles	L2LL	227 original sentences
RUS Dmitrieva and Tiedemann (2021)	Literary texts	L2LL	69,737 original sentences
ITA Tonelli et al. (2016)	Administrative texts	GP	157 original sentences
ITA Brunato et al. (2015)	Children's literature	PLI	1,060 sentence pairs
ITA Brunato et al. (2015)	Educational material	L2LL	1,356 original pairs
**(Semi)Automatic Approach**			
ENG Kauchak (2013)	Wikipedia	GP	167K sentence pairs
ENG Kajiwara and Komachi (2016)	Wikipedia	GP	492,993 sentence pairs
ENG Zhu et al. (2010)	Wikipedia	GP	108,016 sentence pairs
ENG Narayan et al. (2017)	Wikipedia	GP	5,546 original sentences
ENG Woodsend and Lapata (2011)	Wikipedia	GP	14,831 sentence pairs
ENG Botha et al. (2018)	Wikipedia	GP	1,004,944 original sentences
ENG Pavlick and Callison-Burch (2016)	Miscellanea	CS	4.5 million of simplifying paraphrase rules
ITA Tonelli et al. (2016)	Wikipedia	GP	530 original sentences
FRE Brouwers et al. (2014)	Wikipedia	L2LL	72 original sentences
FRE Cardon and Grabar (2020)	Wikipedia	GP	297,494 sentence pairs
ITA Brunato et al. (2016)	Web corpus	GP	63,000 sentence pairs

As it can be seen, the majority of corpora are manually derived and they were developed assuming a human-oriented perspective. Within this group, the only exception is represented by Collados (2013), who created a parallel corpus of 3,000 original and manually simplified sentences for the Spanish language to be used as a pre–processing steps for NLP tasks.

### 2.1. Manual Approach

According to this approach, the simplification process is typically performed by qualified linguists expert in text simplification or professionals (e.g., speech therapists, translators) and it starts from a previously chosen text, which is considered complex for a specific readership and simplified by the expert to improve user's comprehension. The original and simplified versions of this text are then paired either automatically or manually. As anticipated in Section 1, as a universal *native simplified–language speaker* does not exist (Siddharthan, 2014), manually-built corpora differ with respect to the expertise of the “human simplifier.” With the intent of grouping together human simplifiers sharing a common methodology to text simplification, in the literature, it has been drawn the main distinction between two manual simplification strategies: the “structural” and the “intuitive” one, according to Allen's definition (Allen, 2009). The former uses predefined graded lists (covering both word and structural levels) or traditional readability formulas based on shallow proxies of language complexity, the latter is dependent on the professional's intuition on which sentence transformations are needed to reduce the linguistic complexity of a text for a given user, e.g., the author's teaching experience and personal judgments about the comprehension ability of learners. These two strategies have been explicitly taken into account for the collection of Italian and Basque corpora. Specifically, Brunato et al. (2015) compiled two Italian corpora of aligned complex/simple sentences: the *Terence* corpus, a collection of 1,060 pairs of sentences produced by a pool of experts (i.e., linguists and psycholinguists) in the framework of a past EU project Terence^2^ as representative of the “structural” strategy. The experts manually simplified 32 short novels for children aged 7–11 affected by text comprehension difficulties by following a predefined guideline tackling the simplification at three separate textual dimensions, i.e., global coherence, local cohesion and, lexicon/syntax. The intuitive strategy is represented by the so–called *Teacher* corpus, a collection of 24 pairs of original/simplified texts, collected from specialized educational websites that offer free resources for teachers on different textual genres, from famous Italian novels to handbooks for high school on diverse subjects (e.g., history, geography). In this case, texts were simplified by a school teacher who aimed at making them easier–to–read for students, especially L2 learners. Similarly, for the Basque language, Gonzalez-Dios et al. (2017) gathered a collection of documents belonging to the scientific popularization domain manually simplified by a court translator according to easy-to-read guidelines (as representative of the “structural” strategy) and by a teacher based on her/his experience (as representative of the “intuitive” strategy).

For what concerns the textual genre dimension, corpora of newspaper articles are largely predominant. This is the case of the *Newsela* corpus (Xu et al., 2015) which includes a collection of news articles, each one manually simplified at four distinct levels. The multiple simplification versions are a direct consequence of the primary aim of the corpus, which was conceived to help teachers prepare curricula that match the English language skills required at each grade level. A similar purpose is shared by Goto et al. (2015), who simplified a corpus of news for learners of Japanese as a second language. Newswire is also the main genre of the 200 articles contained in the Spanish corpus by Bott and Saggion (2014), which were manually simplified by professionals for people affected by Down's syndrome. Similarly, Klerke and Søgaard (2012) compiled a corpus of aligned original/simplified news intended for adults with cognitive impairment and adult learners of Danish; Caseli et al. (2009) made available a corpus of 104 Brazilian newspaper articles, developed in the framework of the *PorSimples* project, which were manually simplified adopting two different types of approaches to simplification, i.e., *natural* and *strong*, to attend the needs of people (adults and children) with different levels of literacy. Beyond newswire texts, other corpora contain texts from a mix of genres, and for some languages also genre–specific resources have been released covering, e.g., biomedical texts (Grabar and Cardon, 2018), science articles as in

All these examples also highlight that the target reader population is a fundamental factor in driving the construction of ATS corpora. As we introduced at the beginning of this paper, people who need assistive technologies and learners at different levels of proficiency, both in the native and in a second language, are the two main groups of readers targeted by ATS. The first group is addressed for example by the FIRST project (Orasan et al., 2013), a project launched in 2011 aimed at developing tools and resources to assist people with autism spectrum disorders (ASD). A main outcome of the project was a multilingual corpus of 25 texts (for a total of 320 sentences) available in three different languages (English, Spanish, Bulgarian) and covering a wide range of topics, which were manually simplified by professionals (teachers, psychologists, speech and language therapists, and psychiatrists) to improve reading and comprehension of ADS people. The second main group of target readers was taken into account, for example, in the framework of the PorSimples project for the Portuguese language (Aluísio et al., 2008; Caseli et al., 2009) devoted to developing text tools for promoting digital inclusion and accessibility mainly for native language people with low literacy levels. For German, a newspaper corpus compiled by Sauberli et al. (2020) was manually simplified for second language learners at two language levels, B1 and A2 (based on the Common European Framework of Reference for Languages standard), and exploited to test state-of-the-art neural machine translation techniques. More recently, specific attention to children facing problems in reading was paid by Gala et al. (2020), who compiled a corpus of literary and scientific materials available for students in French primary schools and manually simplified them at different linguistic levels, i.e., lexical, morpho-syntactic, syntactic, and discourse. Their final goal was to test the simplified materials with poor-reading and dyslexic children to assess the impact of simplification operations in reducing reading errors.

A survey of very recent works dealing with the collection of parallel corpora also highlights the exploitation of a new manual strategy, in addition to the two main ones discussed so far (i.e., structural and intuitive). It relies on crowd-sourcing techniques to collect human simplified versions of original sentences and so far it has been applied to the English Wikipedia pages. The two most notable corpora obtained in this way are TURKCORPUS (Xu et al., 2016) and ASSET (Alva-Manchego et al., 2020a), differing at the level of rewriting operations adopted to obtain the human simplified version of the original sentences. Another motivation driving the introduction of this new strategy concerns the use of the collected resources as benchmarks to define new, more human-oriented, metrics able to evaluate the ability of ATS systems to generate easy-to-read sentences that not only preserve the original meaning but also sound fluent and simply according to the correlation with human judgments.

### 2.2. (Semi-)Automatic Approach

This second type of approach to building ATS corpora gathers together strategies that even with minor differences allow searching, in a large reference resource, texts which are equivalent in meaning but different at the level of linguistic complexity. The multiple versions of these texts can be written independently, so they are not strictly parallel; they are aligned in a later stage, generally at the sentence level, using word-level (Barzilay and Elhadad, 2003; Nelken and Shieber, 2006; Coster and Kauchak, 2011) or sentence-level (Bott and Saggion, 2011) similarity functions. As already mentioned in the introduction, the most typical example of automatically (or semi-automatically) derived corpora were obtained by aligning articles from the standard and the simple version of the English Wikipedia. It is the case of the corpora described by Kauchak (2013), by Kajiwara and Komachi (2016), and by Zhu et al. (2010) for English. A similar attempt has been pursued by Brouwers et al. (2014), who semi-automatically aligned 20 articles from the French Wikipedia with their equivalents in Vikidia, a small online encyclopedia intended for young readers which gathers more accessible articles than Wikipedia, both in terms of language and content^3^.

Different use of the Wikipedia resource for the construction of monolingual parallel corpora has been shown by Woodsend and Lapata (2011), and more recently by Tonelli et al. (2016) and by Botha et al. (2018). They started from the same assumption that the multiple revisions underlying Wikipedia articles can be used to collect reliable resources for ATS purposes. The resulting corpora are made of aligned sentence pairs where the complex sentence is the one occurring in a previous version of a Wikipedia article and the simple one is the outcome of all edit operations involving a sentence split (Botha et al., 2018), or only those marked by the Wikipedia's contributor as a simplification or grammatical correction (Woodsend and Lapata, 2011; Tonelli et al., 2016).

Beyond Wikipedia data, other text sources were explored. For instance, Narayan et al. (2017) started from the dataset described in Gardent et al. (2017), where each item consists of a set of RDF triples (corresponding to an abstract meaning representation) and one or more texts that verbalize the triples and contain one or more sentences. They used it to automatically create the WEBSPLIT corpus, a very large dataset of 1,066,115 distinct pairs of complex/simple sentences where each distinct complex sentence is associated with multiple (2–7) simpler versions sharing the same abstract meaning representation. The rationale was to segment each verbalization of the same original (complex) sentence into multiple sentences to build a resource useful to be used to train an ATS system able to perform a subset of sentence transformations involved in the simplification process, namely sentence split and rephrase. The construction of a resource containing instances of a single type of simplification rule was Pavlick and Callison-Burch (2016)'s goal, who semi-automatically built the *Simple Paraphrase Database* a corpus of 4.5 million lexical paraphrases devoted to the development of lexical ATS systems.

A further implementation of the automatic approach was proposed by Brunato et al. (2016). To our knowledge, it is the first one not based on Wikipedia and not specifically devised for the English language. Considering the scarcity of a large quantity of aligned data in languages other than English, the authors proposed an approach that does not rely on any kind of pre-existing parallel corpora: this makes such an approach highly scalable and language agnostic. The authors followed the intuition that sentences conveying the same information but with a different level of complexity can be extracted from a large–scale, monolingual corpus of heterogeneous genres and domains, such as the web corpus. According to these premises, they conceived a semi-unsupervised methodology to detect and pair sentences with overlapping lexicon (thus, guaranteeing that the pair had the same meaning) but showing structural transformations of different types. The two sentences of the same pair were then ranked for linguistic complexity, which was calculated according to the score automatically assigned by a readability assessment tool, i.e., the “simple” sentence of the pair was the one assigned with a lower readability score. The approach was tested for the Italian language, resulting in a corpus, named PaCCSS–IT (*Parallel Corpus of Complex-Simple Sentences for ITalian*), which contains about 63,000 pairs of complex/simple sentences.

## 3. An Overview of the Main Simplification Operations Across Corpora

As we mentioned in the introduction, parallel corpora have a strong application value since they primarily serve as training and evaluation resources for ATS systems, being them rule-based or, especially in more recent years, based on deep learning. This means that if an automatic system learns a model of “simple” language from available training corpora, we expect that it would apply it to generate new simplified texts. Therefore, comparing how the complex and the simple version of a sentence vary is crucial to assess the quality of these resources and their suitability for ATS purposes. In this section, we thus take a closer look at the most representative types of transformations occurring in the corpora previously described. To perform this analysis, we moved from the observation that many of the existing parallel resources were annotated with a set of simplification rules aimed at identifying the specific types of linguistic phenomena changing between the original and the simplified version of a sentence. However, as pointed out by Bott and Saggion (2014), these phenomena are not necessarily comparable since the classifications of simplification operations can vary according to language- and genre-specific properties or to the needs of the expected readership. This is the reason why we focus here on a representative set, without the ambition to report an exhaustive list. In particular, we chose to analyse the ones that better fit with the main focus of our investigation, thus considering those rules that have an impact on the morpho-syntactic and syntactic structure of the simplified sentences, and we deliberately paid less attention to the numerous types of transformations affecting the use of words at the lexical level. Indeed, although lexical properties represent a very important and well-investigated aspect in text simplification (Paetzold and Specia, 2017), accounting for them would open an orthogonal but different area of research, with several other variables to be considered, largely inspired by cognitive models on the organization of the mental lexicon, such as word frequency, word length, familiarity, concreteness, imageability, age of acquisition (Cutler, 1983). Moreover, while the English language can rely on large-scale machine readable dictionaries curated by experts where entries are labeled for many of these properties [see, e.g., the Medical Resource Council (MRC) Psycholinguistic Database (Wilson, 1988)], less-resourced languages have to cope with the unavailability, or rather poorer coverage, of such lexical databases; this makes it necessary to supply them with more traditional resources, such as word frequency and word familiarity lists drawn from large corpora. As described in Section 4.2, the only lexical aspect we took into account in this study as a marker of lexical complexity is word frequency considering a representative lexical resource of Italian.

**Split**: breaking down long sentences into shorter ones is probably one of the most studied simplification operations, also from the point of view of its computational treatment (Siddharthan, 2002; Collados, 2013; Narayan et al., 2017). Typical candidates for splitting are coordinate clauses (introduced by coordinating conjunctions, colons, or semicolons), subordinate clauses (e.g., non-restrictive relative clauses, as in the example below), appositive and adverbial phrases. Nevertheless, some real examples detected across ATS parallel corpora showed that human experts do not exploit the split rule as much as expected (Brunato et al., 2015; Xu et al., 2015). A complex sentence may be judged more comprehensible than a simple one, for instance because it contains a subordinate clause that provides the necessary background information to understand the main clause.

O: Mamma Gorilla sembrava completamente distrutta per le cure che dava al suo vivace cuccioletto Tito, **che stava giocando vicino alle grosse sbarre di acciaio che circondavano il recinto**. [lit. Mummy Gorilla looked completely worn out from looking after her lively baby, Tod, **who was playing by the thick steel bars that surrounded the enclosure**.]
S: Mamma Gorilla sembrava proprio distrutta per le cure che dava al suo vivace cuccioletto Tito. *Tito stava giocando vicino alle grosse sbarre di acciaio che erano intorno alla loro area*. [lit. Mummy Gorilla looked completely worn out from looking after her lively baby Tod. *Tod was playing by the thick steel bars that surrounded the enclosure*.]

(Terence corpus, Brunato et al., 2015)

**Merge**: this operation joins two (or more) original sentences into a unique sentence, thus it has the opposite effect of a split. Despite adding more propositions per sentence could make it harder to process (Kintsh and Keenan, 1973), such an operation sometimes allows writers to avoid unnecessary repetition, to clarify the logical order of events with explicit connectives, and to improve sentence variety, with a positive effect on the reader's comprehension.

O: **Gli ebrei debbono consegnare le biciclette. Gli ebrei non possono salire in tram, gli ebrei non possono più andare in auto**. [lit. **Jews have to hand over their bikes. Jewish are not allowed to get in the tram. Jewish are not allowed to drive cars**.]
S: *Gli ebrei non possono più andare in bicicletta, non possono salire in tram e non possono andare in auto*. [lit. *Jews have to hand over their bikes, are not allowed to get in the tram and are not allowed to drive cars*.]

(Teacher corpus, Brunato et al., 2015)

**Reordering**: another possible strategy to simplify texts consists in changing the position of the elements in a sentence, possibly yielding the unmarked order of that language, which is associated with easier comprehension and earlier acquisition (Slobin and Bever, 1982). As shown by the examples here reported, reordering can affect single words, phrases, or entire clauses.

O: **In 1962**, Steinbeck received the Nobel Prize for Literature.
S: Steinbeck won the Nobel Prize in Literature *in 1962*.

(English Wikipedia corpus, Coster and Kauchak, 2011)

O: Aireak hegazkinaren inguruan duen jokabidea zoruak alda dezake, **hegaldia oso baxua denean**. [lit. The soil can change the behavior that the air has around the plane, **when the flight is very low**.]
S: *Hegaldia oso baxua denean* zoruak hegazkinaren inguruko airearen jokabidea alda dezake. [lit. *When the flight is very low*, the soil can change the behavior that the air has around the plane.]

(Basque corpus, Gonzalez-Dios et al., 2017)

O: Ringraziandola per la sua cortese attenzione, **resto in attesa di risposta**. [lit. Thanking you for your kind attention, **I look forward to your answer**.]
S: *Resto in attesa di una risposta* e ringrazio vivamente per l'attenzione. [lit. *I look forward to your answer* and I thank you greatly for your attention.]

(PaCCSS–IT corpus, Brunato et al., 2016)

**Insert**: the process of simplification may even result in a longer sentence because of the insertion of words or phrases that provide additional information to the original sentence and possibly reduce the inference load of a text. Despite the cognitive literature suggests reducing the inference load of a text, especially when it targets less skilled or low-knowledge readers (Ozuru et al., 2009), it is difficult to predict what an author will add to the original sentence to make it clearer. The sentence can be elliptical, i.e., syntactically compressed, and the difficulty depends on the ability to retrieve the missing arguments, which are then made explicit as a result of the simplification. The following examples show a case of insertion of the main verb and a subject, respectively. The insertion of a subject has to be intended as the transformation of a covert subject into a lexical noun phrase, which is an option available in null-subject languages (e.g., Italian).

O: Escuela Segura, un compromiso municipal con la proteccioń integral de los escolares. lit. [Safe School: a municipal promise for the full protection of school kids.]
S: Escuela Segura *es* un programa municipal para la proteccioń de los escolares. [lit. Safe School *is* a municipal promise for the full protection of school kids.]

(Spanish corpus, Bott and Saggion, 2014)

O: Curiosa com'era, si avvicinò per osservarla meglio, prima timidamente, poi con più coraggio. [lit. Curious as she was, (she) moved closer to watch it better, shyly at first, than more courageously.]
S: Curiosa com'era, *Ernesta* si avvicinò per guardarla meglio, prima con paura, poi con più coraggio. [lit. Curious as she was, *Ernestine* moved closer to watch it better, timidly at first, than more courageously.]

(Terence corpus, Brunato et al., 2015)

**Delete**: removing redundant information has proven to be another effective strategy to simplify a text. Like insertion, it is difficult to predict which words could be removed, although we can predict that simplified sentences would contain fewer adjunct phrases (e.g., adverbs or adjectives). In null-subject languages, a particular case of deletion is the substitution of a lexical noun phrase subject with a covert pronoun, especially when the latter points to a referent which is highly prominent in the context, as shown by the last example.

O: The crust and **underlying relatively rigid** mantle make up the lithosphere.
S: The crust and mantle make up the lithosphere.

(English Wikipedia corpus, Coster and Kauchak, 2011)

O: Poi la nuvoletta aggiunse, con molta tristezza, che purtroppo **lei** stava partendo, come ogni anno. [lit. Then the little cloud said, with much sadness, that unfortunately **she** was leaving, like every year.]
S: La nuvoletta, un po' triste, disse che stava partendo, come tutti gli anni. [lit. The little cloud, a bit sad, said that (she) was leaving, like every year.]

(Terence corpus, Brunato et al., 2016)

**Transformations**: the macro-class of sentence transformations (or sentence changes) is articulated into more fine-grained operations representative of specific linguistic phenomena, which affect the lexical, morpho-syntactic, or syntactic structure. Here follows a list of major sentence transformations:

–* Lexical substitution*: the substitution of a complex word with an easier synonym is a feasible way to reduce the linguistic complexity of a text. Much research in ATS has been done on lexical simplification trying to automatize this process, e.g., by relying on electronic resources, such as WordNet (DeBelder and Moens, 2010), word frequency lists (Drndarevic et al., 2012) or simpler paraphrases (Kriz et al., 2018)^4^. However, corpus analysis highlighted that a complex word can be substituted by a multi-word paraphrase rather than a synonym or even explained by a gloss, especially for the technical terms.

O: Dopo la scoperta del cadavere di Lily Kimble, la polizia comincia a interessarsi al caso. Dal canto loro, i Reed capiscono che finchè l'assassino non sarà **al fresco**, la loro vita sarà in pericolo. [lit. After the discovery of Lily Kimble's body, the police begin to take an interest in the case. For their part, the Reeds realize that until the killer is not **in the pen**, their lives are in danger.]
S: Dopo la scoperta del cadavere di Lily Kimble, la polizia comincia a interessarsi al caso. Dal canto loro, i Reed capiscono che finchè l'assassino non sarà *in carcere*, la loro vita sarà in pericolo. [lit. After the discovery of Lily Kimble's body, the police begin to take an interest in the case. For their part, the Reeds realize that until the killer is not *in jail*, their lives are in danger.]

(Simpitiki-Admin corpus, Tonelli et al., 2016)

O: Poiché era indeciso su quale fosse il bidone giusto, chiese ad un **passante** di indicargli il bidone dove buttare la carta. [lit. Since he was unsure about which dustbin was the right one, he asked to a **passer-by** to point him the dustbin to throw paper.]
S: Poiché non sapeva quale fosse il bidone giusto, chiese ad un *signore che passava* dove era il bidone per la carta. [lit. Since he didn't know which dustbin was the right one, he asked to a *man who was walking* where the paper dustbin was.]

(Terence corpus, Brunato et al., 2015)

–*Anaphoric phenomena*: under this class, there were marked transformations involving the substitution of a referential pronoun in the original sentence with its full lexical antecedent (a definite noun phrase or a proper noun) or vice versa. As shown by the examples that follow, in several cases a transformation at one level triggers rearrangements at other levels of the sentence, which are necessary for the grammatically of the simplified output. For instance, replacing a direct or indirect object pronoun (which is preverbal in some languages like Italian) with its full lexical antecedent not only changes the grammatical category of the element but also affects syntactic order, since full nominal objects follow the verb.

O: Il passante gli spiegò che, per arrivare al bidone, **doveva contare ben 5 bidoni a partire dal semaforo**. [lit. The passer-by explained him that, to get to the dustbin, **he had to count exactly 5 dustbins starting from the traffic light**.]
S: Il signore spiegò a Ugolino che *doveva contare 5 bidoni a partire dal semaforo*, per arrivare al bidone della carta. [lit. The man explained Little Hugh that *he had to count 5 dustbins starting from the traffic light* to get to the wastepaper dustbin.]

(Terence corpus, Brunato et al., 2015)

O: Anche Federico Fellini, all'epoca ancora giovane e sconosciuto, aiuterà **Aldo Fabrizi** nella sceneggiatura. [lit. Also Federico Fellini, still young and little known at that time, will help **Aldo Fabrizi** in the script.]
S: Anche Federico Fellini, all'epoca ancora giovane e sconosciuto, *lo* aiuterà nella sceneggiatura. [lit. Also Federico Fellini, still young and little known at that time, will help *him* in the script.]

(Simpitiki-admin corpus, Tonelli et al., 2016)

–*Nominalization phenomena*: these transformations target a nominalization (or a support verb construction), which is replaced by the simple verb from which it derives, or conversely, a simple verb which is changed into a nominal phrase headed by the corresponding derivative noun.

O: Il giorno **della partenza**, i bambini salutarono i loro genitori durante la colazione. [lit. On the day of their **parents' departure**, the children said their goodbyes to their parents over breakfast.]
S: Il giorno *in cui i genitori partirono*, i bambini li salutarono durante la colazione. [lit. The day *that their parents left*, the children said them goodbye over breakfast.]

(Terence corpus, Brunato et al., 2015)

O: Un computer simile è presente nel film Pixar Wall-e: il computer della nave spaziale axiom **si chiama** AUTO e governa la nave assieme al capitano. [lit. A similar computer is present in the Pixar Wall-e movie: the axiom spacecraft computer **is called** AUTO and rules the ship with the captain].
S: Un computer simile è presente nel film Pixar Wall-e: il computer della nave spaziale axiom *di nome* AUTO e governa la nave assieme al capitano. [lit. A similar computer is present in the Pixar Wall-e movie: the axiom spacecraft computer *with name* AUTO and rules the ship with the captain].

(Simpitiki-Admin corpus, Tonelli et al., 2016)

–* Voice*: as a result of simplification, a passive sentence may be converted into an active one or vice versa. The former transformation is more expected when humans simplify a text because passive sentences are considered as more complex according to language acquisition data in typical (Maratsos, 1974) and atypical populations, e.g., deaf children (Volpato, 2010). Plain language guidelines also recommend preferring active than passive voice^5^. Yet, the “passivization” rule may still be productive in specific textual typologies like administrative texts, where the author of the simplification can prefer not only to keep but even to insert, a passive, to avoid more unusual syntactic constructs (such as impersonal sentences).

O: **Se trata de un proyecto (…) que coordina el trabajo (…) de las delegaciones municipales de Educacioń y Seguridad**. [lit. **It consists of a project that coordinates the work of the city's education and security delegations**.]
S: *El proyecto está coordinado por las delegaciones municipales de Educacioń y Seguridad*. [lit. *The project is coordinated by the city's education and security delegations*.]

(Spanish corpus, Bott and Saggion, 2014)

O: **Rinvia, quindi, il seguito dell'esame ad altra seduta**. [lit. **He/she postpones, thus, the follow-up examination to other hearing** .]
S: *Il seguito dell'esame viene rinviato ad altra seduta*. [lit. *The follow-up examination is postponed to other hearing*.]

(PaCCSS–IT corpus, Brunato et al., 2016)

–* Verbal features*: The simplification of a text can also alter the distribution of verbal features (such as mood, tense, and person), especially in languages with a rich inflectional paradigm. These features indeed are involved in text complexity as proven by literature on readability assessment, in which verbal features appear upon the variables that discriminate between easy- and difficult-to-read texts (Attardi et al., 2009; Dell'Orletta, 2009; Bautista et al., 2011; Dell'Orletta et al., 2011; François and Fairon, 2012; Narayan and Gardent, 2014). Moreover, for some categories of readers (e.g., second-language learners) some inflections are more difficult to master; thus simplified texts targeting these readers should exhibit more common and less literary tenses than those used in the original texts (Brouwers et al., 2014).

O: Tali elementi **dovranno** supportare e giustificare le scelte progettuali operate. [lit. Such elements **will have** to support and justify project decisions].
S: Tali elementi *devono* supportare e giustificare le scelte progettuali operate. [lit. Such elements *have* to support and justify project decisions.]

(Simpitiki-Admin corpus, Tonelli et al., 2016)

## 4. A Two-Level Comparison

As described in Section 2, the majority of works on ATS corpora have been devoted to study text simplification with a special interest for the target audience and the expertise of the human simplifier, as well as for the influence of the textual genre. Less attention has been paid to inspecting whether and to what extent the methodology adopted to build ATS resources can affect the structure and the linguistic characteristics of the simplified sentences. To shed light on this under-investigated perspective, in this section we present a methodology based on a fine-grained linguistic analysis aimed at understanding (1) in which respect complex and simple/simplified texts vary and (2) whether and how the observed changes may depend on a manual or a (semi-)automatic approach.

The analysis has been carried out at two levels: concerning the distribution of the simplification operations described in the previous section and of multi-level linguistic features automatically extracted from texts and modeling a wide range of morphosyntactic and syntactic phenomena involved in sentence complexity. In the first case, the aim is to figure out whether some operations are specific only to a given building approach or the same type of simplification operations occurs independently. This would suggest that some sentence transformations should be considered as more “fundamental” to yield a simpler text. Consequently, if an automatic text simplification system learns a model of “simple” language from corpora containing these transformations, we expect that it would apply them to newly generated texts. Secondly, the analysis of the automatically extracted linguistic phenomena is meant to detect similarities or differences between the original and simplified sentences: this type of information may also represent a valuable contribution to evaluate the quality of a resource to be used in real ATS scenarios.

For both levels of analysis, we chose to focus on Italian since this is the only language, except English, for which there are quite large resources derived through the manual and automatic approach. Specifically, we relied on three corpora: the *Terence* and *Teacher* corpora, representative of the manual approach, and more specifically of the “structural” and the “intuitive” approach, and PaCCSS-IT, as representative of the automatic approach. However, the whole methodology is in principle transferable to multiple languages since it relies on sentence transformations shared by many ATS resources, as discussed in Section 3, and on a multi-lingual approach to the automatic extraction of linguistic phenomena, as it is detailed in Section 4.2.

### 4.1. Distribution of Simplification Operations

As described in Brunato et al. (2016), the approach devised to create PaCCSS-IT was aimed at collecting sentence pairs sharing the same meaning but with a few structural transformations possibly affecting the level of linguistic complexity. To control for meaning preservation, the pairs were selected to share many of their words, namely: the same lemmas tagged as nouns, verbs, and adverbs for what concerns open-class categories, and the same personal pronouns and negative adverbs, for what concerns closed-class categories. Given these strict requirements, some of the simplification operations described in Section 3 are not allowed in PaCCSS-IT by definition: for example, since the alignment between the complex and simple sentence has a correspondence 1:1, the operations involving the splitting and merging of sentences are not possible; likewise, transformations involving the replacement of a verb with a deverbal noun (i.e., nominalization) do not occur since the aligned sentences must contain the same nouns and verbs. To allow a comparative analysis between the manually and automatically collected corpora, we thus selected only the subset of rules potentially occurring in all corpora—which correspond to the macro-level operations—and used them to manually annotate the whole *Terence* and *Teacher* corpora and a comparable portion of PaCCSS-IT corresponding to a subset of 921 paired sentences. All corpora were annotated by two undergraduate students in computational linguistics, who received preliminary training lessons on the simplification rules covered by the annotation tagset. All their annotations were verified by two of the authors of the paper.

**Figure 1:**
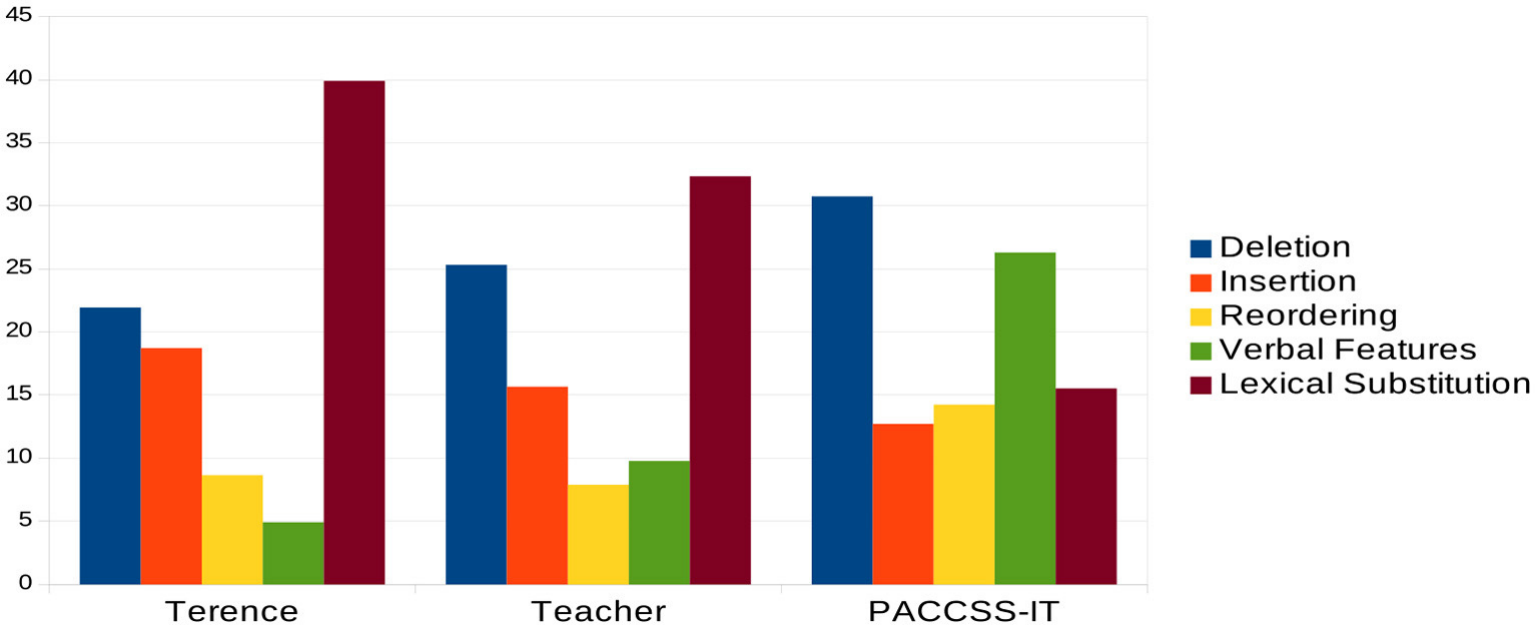
Distribution of simplification operations across corpora.

Figure 1 reports the distribution of simplification operations in the examined corpora. In line with the criteria adopted to build PaCCSS-IT, it can be noted that the automatic approach is mostly characterized by structural transformations (i.e., deletions, insertions, and reordering), which cover almost 60% of the whole amount of operations. On the contrary, operations involving the substitution of words are more frequently exploited in the manual process of sentence simplification (*Terence*: 39.89%; *Teacher*: 32.33%; PaCCSS-IT: 15.52%). Among the operations modifying the syntactic structure, the deletion and the insertion of linguistic material (words or phrases) have a similar frequency across the three corpora. As expected, removing redundant information turned out to be the most frequent sentence transformation (*Terence*: 21.94%; *Teacher*: 25.32%; PaCCSS-IT: 30.74%). Interestingly, the automatic approach intercepts a wider set of simple sentences where phrases and words have been reordered thus possibly showing a more canonical word order which is easier to process (Diessel, 2005; Futrell et al., 2015). A further operation that clearly differentiates the automatic approach from the manual one affects verbal features. Given that the complex and the simple sentences in PaCCSS-IT share the same verb lemmas, the higher percentage of this operation (26.30%) concerns only transformations of the same lemma which changes concerning, e.g., mood, tense, person, and verbal voice. Notably, this is in line with the predominant role played by structural transformations characterizing the automatic approach: for example, the passive/active alternation or the occurrence of implicit vs. explicit moods imply syntactic modifications of the whole sentence.

### 4.2. Distribution of Linguistic Phenomena

While in the previous section we focused on the comparison between the manual and automatic approach concerning the distribution of simplification rules, here we examine the distribution of a wide set of linguistic phenomena characterizing the complex and simple sentences of each corpus. To conduct this analysis we rely on the methodology of “linguistic profiling,” an NLP-based framework of analysis in which a large number of counts of linguistic features extracted from linguistically annotated corpora are used as a text profile and can then be compared to average profiles of texts (or groups of texts) to identify those that are similar in terms of the profiled features (van Halteren, 2004; Montemagni, 2013). This methodology, which is rooted in the seminal works by Douglas Biber who first introduced the multidimensional approach to linguistic analyses of genre variation (Biber, 1993, 1995), has been successfully used in a variety of application scenarios, all focused on the “form” rather than the content of texts: from automatically modeling the developmental patterns in child language acquisition (Lu, 2009; Lubetich and Sagae, 2014) and the evolution of written language competence in school learners' (Weiss and Meurers, 2019; Miaschi et al., 2021), to the prediction of behavioral and cognitive impairments based on the detection of relevant linguistic markers from clinical tests (Roark et al., 2007; Prud'hommeaux et al., 2011); also, in the context of computational sociolinguistics, it has been used for studying variations related to the social dimension of language (Nguyen et al., 2016) or for modeling stylometric characteristics of authors or author groups (Daelemans, 2013).

For our analysis, we relied on Profiling-UD (Brunato et al., 2020), a tool recently introduced that implements the assumptions of linguistic profiling and specifically conceived for corpora annotated according to the Universal Dependencies (UD) framework^6^. UD is an ongoing project aimed at developing corpora with a cross-linguistically consistent annotation for many languages, to facilitate multilingual parser development, cross-lingual learning, and parse research from a language typology perspective (De Marneffe et al., 2016). The choice of relying on UD-style annotation makes the process of feature extraction language-independent, as similar phenomena are annotated according to a common annotation scheme at morpho-syntactic and syntactic levels of analysis. The tool performs a two-stage process: linguistic annotation and linguistic profiling. The annotation of the text(s) is carried out by UDPipe (Straka et al., 2016) using the available UD model(s) for the input language. The automatically annotated text(s) are used as input to the further step, performed by the linguistic profiling component defining the rules to extract and quantify the formal properties.

Profiling-UD allows the computation of a wide set of features encoding a variety of morpho-syntactic and syntactic properties of text, which are reliable predictors in a variety of scenarios, from stylometric analyses to genre classification. For our specific purposes, we considered only a subset of them, namely those that have been used in the literature to assess the readability level of texts (Collins-Thompson, 2014) or to investigate which of these features correlate with human judgments on sentence complexity (Brunato et al., 2018). Specifically, they range from superficial ones, such as the average length of words and sentences, to morpho-syntactic information concerning the distribution of parts-of-speech (POS)^7^ and the inflectional properties of verbs, to more complex aspects of syntactic structure deriving from the whole parse tree and specific sub-trees (e.g., subordinate clauses). A sub-set of features, which we considered particularly relevant for this study, is reported in Table 2 where they are grouped into main linguistic phenomena and distinguished according to the level of annotation from which they derive.

**Table 2:** Overview of the linguistic features used for linguistic profiling.

**Level of annotation**	**Linguistic feature**
Raw Text	**Raw text properties**
	Sentence length
	Word length
Vocabulary	**Vocabulary richness**
	Type/Token ratio for words and lemmas
POS tagging	**Morphosyntactic information**
	Distribution of UD and language–specific POS
	Lexical density
	**Inflectional morphology**
	Inflectional morphology of lexical verbs and auxiliaries
	**Verbal predicate structure**
	Distribution of verbal heads and verbal roots
	Verb arity and distribution of verbs by arity
	**Global and local parsed tree structures**
	Depth of the whole syntactic tree
	Average length of dependency links and of the longest link
	Average length of prepositional chains and distribution by depth
	Clause length
Dependency parsing	**Order of elements**
	Relative order of subject and object
	**Syntactic relations**
	Distribution of dependency relations
	**Use of subordination**
	Distribution of subordinate and principal clauses
	Average length of subordination chains and distribution by depth
	Relative order of subordinate clauses with respect the main clause

**Figure 2:**
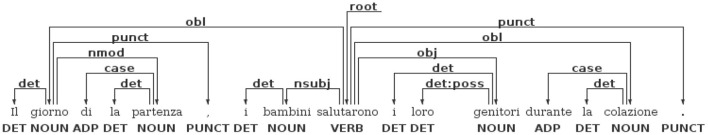
A sentence from the corpus linguistically annotated in the UD-format.

As an example, we report in Figure 2, a graphical representation of the output of the linguistic annotation in UD format for a sentence of the *Terence* corpus. By applying Profiling-UD on this input sentence, we can observe, for instance, that the sentence contains 16 tokens and these tokens are on average 4.93 characters long. Concerning the distribution of POS, there is 31.25% of nouns, 6.25% of verbs, and 37.5% of determiners, among others. At syntactic level, since we only have one verbal root represented by the main predicate [i.e., *salutarono*, (greeted)], the arity value is 4 corresponding to the four dependents attached to the head [i.e., *giorno* (day) and *colazione* (breakfast), both bearing the role of oblique modifiers, and *bambini* (children) and *genitori* (parents) with the role of subject and object, respectively]. Moreover, the average length of dependency links is 2.38 and the longest link has a value of 7, which corresponds to the number of words separating the dependent *giorno* from its head *salutarono*.

In addition to the set of features extracted by Profiling-UD, we calculate some extra ones characterizing the lexical profile of a sentence, in terms of the percentage distribution of words belonging to the *Basic Italian Vocabulary* (*BIV*) by DeMauro (2000). This is a reference lexical resource for contemporary Italian covering about 7,000 words considered as highly familiar to Italian native speakers. As described in Chiari and De Mauro (2014), VdB derives from a combination of statistical criteria used to select lemmas (both grammatical and content words) mainly based on a frequency list of written Italian, which was subsequently enriched with a frequency list of spoken Italian, and experimental evaluations with primary school pupils. Since its first edition, the final resource is internally subdivided into three usage repertories: “fundamental words” (*FO*), i.e., highest frequency words that cover about 90% of all written and spoken texts), “high usage words” (*HU*), i.e., about 6% of the subsequent high-frequency words) and “high availability words” (*HA*), relatively lower frequency words referring to everyday life whose detection is not based on textual statistical resources but is derived from psycholinguistic insights experimentally verified.

Using Profiling-UD, we thus proceeded to automatically parse all texts of our corpora up to the level of syntactic annotation and to convert them into a rich feature-based representation. For each feature, also including the distribution of lexicon of the *Basic Italian Vocabulary*, we then assessed whether the average distribution in the relative corpus changes significantly between the original and the simplified sentences using a non-parametric statistic text, i.e., the Wilcoxon signed-rank test. Tables 3, 4 report the distribution of an excerpt of features whose variation between complex and simple sentences resulted to be statistically significant for at least one of three considered corpora according to the Wilcoxon signed-rank test.

**Table 3:** Distribution of the raw text, lexical, and morpho-syntactic features in the complex and simple set of sentences for the three corpora.

**Feature**	**Terence**			**Teacher**			**PaCCSS–IT**		
	**Compl**	**Simp**	**Diff**	**Compl**	**Simp**	**Diff**	**Compl**	**Simp**	**Diff**
**Raw text features**									
Sentence length	19.92	18.61	**1.31**	21.25	18.56	**2.70**	8.97	8.0	**0.97**
Word length	4.89	4.80	**0.09**	4.74	4.70	0.04	4.70	4.54	**0.16**
**Lexical features**									
% BIV	75.59	77.31	**−1.72**	78.53	77.77	0.75	72.19	77.08	**−4.88**
% FO	78.14	79.82	**−1.67**	80.21	82.73	**−2.51**	75.03	75.76	**−0.73**
% HU	13.08	12.15	**0.93**	11.98	9.68	**2.30**	20.19	19.82	**0.37**
% HA	8.77	8.03	**0.74**	7.81	7.60	0.21	4.78	4.42	**0.36**
Type/Token ratio	0.942	0.941	-0.001	0.921	0.913	0.008	0.97	0.99	**−0.02**
**Morpho–syntactic features**									
**Morpho–syntactic information**									
Adjectives	5.87	5.97	−0.01	5.34	5.11	0.23	5.74	7.90	**−2.15**
Adverbs	6.82	6.97	−0.15	7.62	6.73	0.89	12.26	9.95	**2.31**
Articles	8.79	8.73	0.07	8.24	8.69	−0.45	11.04	12.71	**−1.67**
Conjunctions—coordinating	3.57	3.76	**−0.19**	3.98	4.72	**−0.74**	2.66	3.45	**−0.79**
Conjunctions—subordinating	1.75	2.16	**−0.41**	1.73	1.09	**0.64**	0.32	0.30	0.02
Prepositions	13.31	12.50	**0.81**	10.77	10.51	0.25	5.98	6.21	**−0.23**
Pronouns	5.33	5.04	**0.28**	17.69	17.15	**0.54**	7.23	4.14	**3.09**
Pronouns—relative	0.87	0.81	0.06	0.85	0.28	**0.57**	0.27	0.1	**0.17**
Pronouns—clitic	2.78	2.61	**0.17**	5.25	2.74	**2.51**	2.47	1.60	**0.87**
Punctuation	11.57	11.54	0.03	15.53	15.52	0.01	20.5	15.13	**5.36**
Numbers	1.07	0.91	**0.15**	2.25	2.47	−0.22	2.25	2.47	**−0.22**
Lexical density	0.59	0.60	**−0.00**	0.58	0.62	**−0.04**	0.61	0.60	**0.00**
**Inflectional morphology**									
Indicative mood	61.23	64.4	**−3.17**	57.14	70.87	**−13.73**	68.14	68.31	**−0.17**
Participial mood	6.95	4.63	**2.32**	3.95	2.84	1.11	3.65	2.42	**1.23**
Gerundive mood	3.44	2.62	**0.83**	1.56	–	**1.56**	0.46	0.04	**0.42**
Infinitive mood	15.98	17.64	**−1.66**	22.1	19.67	**2.43**	12.04	11.65	0.39
Subjunctive mood	1.00	0.57	**0.42**	0.58	–	**0.58**	0.78	0.05	**0.73**
Conditional mood	0.19	0.12	0.07	0.84	0.18	0.66	3.34	0.001	**3.33**
Present tense	6.21	4.74	**1.47**	43.31	90.19	**−46.87**	79.18	80.91	**−1.73**
Imperfect tense	50.66	52.97	**−2.31**	16.39	0.82	**15.57**	2.89	4.29	**−1.40**
Past tense	40.98	39.97	**1.01**	27.45	–	27.45	1.33	1.57	**−0.24**
2 person, singular	0.44	0.51	−0.07	2.77	0.37	**2.4**	0.60	0.44	**0.15**
3 person, singular	64.9	66.09	−1.19	48.59	53.31	−4.72	62.31	58.13	**4.18**
1 person, plural	–	0.09	–0.09	2.95	4.13	−1.18	1.51	1.84	**−0.33**
2 person, plural	–	–	–	0.42	0.32	0.10	0.30	0.19	**0.11**
3 person, plural	18.69	19.14	−0.45	13.86	16.55	−2.69	8.12	7.83	**0.28**

*Statistically significant variations with respect to the Wilcoxon signed-rank test at p < 0.05 are bold*.

**Table 4:** Distribution of the syntactic features in the complex and simple set of sentences for the three corpora.

**Feature**	**Terence**			**Teacher**			**PaCCSS–IT**		
	**Compl**	**Simp**	**Diff**	**Compl**	**Simp**	**Diff**	**Compl**	**Simp**	**Diff**
**Syntactic relations**									
Subjects	6.37	6.87	**−0.50**	5.25	6.71	**−1.46**	7.65	6.94	**0.71**
Objects	4.77	5.12	**−0.34**	4.93	4.92	0.01	1.82	1.90	**−0.07**
Subjects—passive	0.20	0.15	0.04	0.17	0.08	0.09	0.66	0.95	**−0.29**
**Use of subordination**									
Subordinate clauses	51.86	51.41	**0.45**	53.08	47.35	**5.73**	50.083	50.078	**0.005**
Depth of “chains” of subord.	0.39	0.41	**−0.03**	0.39	0.27	0.12	0.05	0.06	**−0.001**
Post-main subordinates	40.54	43.42	**−2.87**	42.27	27.98	**14.29**	3.28	4.17	**−0.89**
**Global and local parsed tree structure**									
Parse tree depth	5.80	5.56	**0.24**	5.10	4.46	**0.64**	2.85	2.70	**0.15**
Dependency links length	2.07	2.03	**0.04**	2.29	2.12	**0.17**	1.76	1.63	**0.13**
Length of the longest link	8.01	7.48	**0.53**	9.24	7.32	**1.93**	3.81	3.31	**0.5**
Verbal arity	1.93	1.95	−0.02	1.85	1.91	−0.05	2.09	2.08	**0.01**
Depth of prepositional “chains”	1.06	1	**0.06**	0.90	0.91	−0.01	0.44	0.41	**0.02**
**Order of elements**									
Pre-verbal subjects	71.07	71.35	−0.28	51.16	61.71	**−10.55**	50.58	43.85	**6.73**
Post-verbal subjects	9.59	11.32	−1.73	16.62	15.51	1.11	15.36	14.37	**0.99**
Pre verbal objects	5.72	5.54	0.17	8.29	3.24	**5.05**	2.03	1.38	**0.65**

*Statistically significant variations with respect to the Wilcoxon signed-rank test at p < 0.05 are bold*.

As a general remark, we can observe that *Teacher* is the corpus with the highest difference between the values of features characterizing the original and simplified sentences. Namely, the average difference between the two versions of the corpus is 4.14 considering the features extracted from raw text, vocabulary, and morpho-syntactic level of annotation, and 2.94 for what concerns the features referring to syntactic phenomena. On the contrary, these differences drop to 1.18 and 0.80 for the sentences contained in PaCCSS-IT, and to 0.72 and 0.52 for the *Terence*'s sentences. This represents the first evidence that the intuitive manual approach yields the sharpest linguistic differences among the considered approaches. However, the amount of features whose value varies significantly between the two counterparts is higher in the corpus automatically built, meaning that, differently from the two types of manual approaches, this method intercepts a large variety of linguistic phenomena that make a sentence easier to read.

If we go more into detail, it can be noted that the simple sentences of each corpus are shorter, in terms of the average number of tokens per sentence. This could be expected since sentence length has been considered as a shallow proxy of sentence complexity and is widely used by traditional readability assessment formulas. The different average length between the original and the simple sentence is also influenced by textual genre: while previous studies on genre variation have shown that narrative prose is characterized by longer sentences (see e.g., Biber, 1995 among others), sentences from the web tend instead to be shorter (Santini, 2007). It follows that the sentences contained in *Terence* and *Teacher* were originally longer and thus the effect of simplification is much more evident. Conversely, the original sentences in PaCCSS-IT already had an average length that is much lower than the average sentence length of the Italian language (i.e., 20–25 tokens) and thus were not greatly modified concerning this parameter. Among raw text features, the average length of words appears to be less concerned with sentence simplification. The three corpora do not vary greatly and length variation of *Teacher*'s words results to be not statistically significant.

Focusing on lexical features, we can see that the use of a more frequent lexicon in simple sentences mostly characterizes PaCCSS-IT and *Terence*. In particular, the percentage distribution of all unique words (types) in the *Basic Italian Vocabulary* (*BIV*) increases in the collection of simple sentences except the *Teacher* corpus, for which the distribution does not change significantly. This is specifically the case of the simple sentences collected with the automatic approach adopted in PaCCSS-IT, which have a higher percentage of BIV concerning the corresponding original sentences (almost 5%). Note that according to the strategy devised to automatically build this resource, such an increase of simple lexicon is mainly concerned with the substitution or the insertion of content and functional words annotated with a Parts-Of-Speech not shared by the original/simple pair (which, we recall here, are necessarily nouns, verbs, numerals, personal pronouns, and negative adverbs). For instance, the following pair contains only a minimal variation, affecting the adverb of time (“conclusivamente”), which is substituted with a more frequent one, with the same meaning, but contained in the BIV.

O: Propone **conclusivamente** di esprimere parere favorevole. [lit. He suggests *eventually* giving a favorable opinion]
S: Propone *infine* di esprimere un parere favorevole. [lit. He suggests *lastly* giving a favorable opinion.]

However, if we focus on the internal classification of BIV into the usage repertories of “fundamental” (FO, very frequent words), “high usage” (HU, frequent words) and “high availability” (HA, relatively lower frequency words referring to everyday life), the simplified sentences contained in the *Teacher* corpus report the highest increase of fundamental lexicon.

The approach adopted in the construction of PaCCSS-IT influences also the distribution of morpho-syntactic characteristics deriving from linguistic profiling. Specifically, while the frequency of nouns and verbs is necessarily the same, the way nominal and verbal modification are expressed changes in the complex and simple version of the pairs: simple sentences have more adjectives, articles, and determiners, but fewer adverbs, punctuation marks, and pronouns. Among the latter, clitic pronouns are much more frequent in the original than in the simplified version (2.47 vs. 1.60). A possible explanation, which is consistent with qualitative observations on the corpus is that, in many cases, a sentence with an impersonal verb construction introduced by a clitic pronoun, is paired with a simple one expressing the same meaning but with a personal verb form, as in the following example.

O: Non **si può fare** di ogni erba un fascio. [lit. **It is not possible** to bundle everybody together in one big bunch.]
S: Però non *possiamo fare* di tutta l'erba un fascio. [lit. But *we can't bundle* everybody together in one big bunch.]

On the contrary, at the level of POS distribution, the differences between the original and simplified sentences contained in the corpora manually simplified are less sharp. Interestingly, the main exception is represented by the *Teacher* corpus and it affects the distribution of clitic pronouns. With this respect, this corpus shares a similar tendency with the one automatically derived, that is a very consistent drop in the use of clitic pronouns, which is even sharper. Again, this can be due to an editing operation changing an impersonal with a personal form but also to the insertion of full lexical items that are substitute of clitics as in the following example, where the two instances of the pronoun *ci* (lit. “us”) were substituted with two different lexical referents:

O: Non poter mai andar fuori mi opprime, e ho una gran paura che **ci** scoprano e **ci** fucilino. [lit. Never being able to go outside oppresses me, and I'm so afraid that they discover **us** and shot **us**].
S: E' triste non andare fuori. Ho una gran paura delle SS: possono scoprire *il rifugio* e uccidere *tutti noi*. [It's sad not to go outside. I'm so afraid of the SS: they can find out about *the shelter* and kill *all of us*.]

Interestingly, among the characteristics extracted from the morpho-syntactic level of annotation, those concerning the inflectional morphology of verbs undergo the main changes. In this case, the main differences can be observed in the *Teacher* corpus, which in its simplified version contains a higher percentage of verbs at the indicative mood and the present tense, and conversely a lower amount of verbs in their imperfect and past tense. Variations of the verbal morphology also occur at the level of person and number of verbs: *Teacher* simplified sentences contain more verbs at the third singular person, at the first and at the third plural person, than their original counterparts.

When we consider the syntactic features, we can observe that all corpora are characterized by noteworthy changes. Among the considered syntactic relations, subjects are more frequent in the simplified sentences of all corpora even if this is, in particular, the case of the corpus representative of the intuitive simplification. This is in line with what was observed concerning the insertion of explicit arguments that allow reducing the inference load of null-subject sentences. Consider for example the following excerpt where the nominal subject *Ernesta* was inserted in the simple counterpart of the original sentence:

O: Curiosa com'era, si avvicinò per osservarla meglio, prima timidamente, poi con più coraggio. [lit. Curious as she was, (she) moved closer to watch it better, shyly at first, than more courageously.]
S: Curiosa com'era, *Ernesta* si avvicinò per guardarla meglio, prima con paura, poi con più coraggio. [lit. Curious as she was, *Ernestine* moved closer to watch it better, timidly at first, than more courageously.]

Similar observations hold for the distribution of direct objects, even if in this case *Teacher* is the only corpus where they are almost stable. According to Profiling-UD, the distribution of subordinate clauses is calculated as the percentage distribution of main vs subordinate clauses, where the latter are identified based on the UD guidelines that distinguish four different types^8^. We considered this feature since the use of subordination is a broadly studied marker of structural complexity, for example for text simplification purposes (Bott and Saggion, 2014). This is particularly the case of post-verbal subordinate clauses that, according to Miller and Weinert (1998), are easier to read than subordinates preceding the main clause. However, if this is confirmed in *Terence* and PaCCSS-IT, an opposite trend can be observed in the *Teacher* corpus, which are characterized by a lower amount of post-verbal subordinates.

The set of features intercepting the global and local syntactic structure of the sentence has been considered since it includes aspects typically related to length factors and correlate with processing difficulty (Frazier, 1985). This is the case of parse tree depth, which can be indicative of increased sentence complexity as stated by, to mention only a few, Yngve (1960) and Gibson (1998). Both Lin (1996) and Gildea and Temperley (2010) showed that the syntactic complexity of sentences can be predicted with measures based on the length of dependency links since long-distance constructions cause cognitive load. As Table 4 shows, in all three corpora the values of these features tend to decrease. This trend concerns specifically the *Teacher* corpus and the minimization of dependency links, and in particular of the longest one in the sentence. The feature was computed as the linear distance between the head and its dependent in terms of tokens. Simple sentences tend to contain shorter dependency links thus increasing the capacity of working memory (Miller, 1956).

The relative order of subject and direct object with respect to the verb has been shown to be harder to process especially in free word-order languages. The position of these core verb arguments is a language-specific property typically connected with “marked” or “unmarked” word orders and thus highly related with sentence complexity or “abnormality,” to put in Haspelmath's words (Haspelmath, 2006). In addition, according to the “adaptability hypothesis,” some linguistic properties systematically lead to varying processing behavior in typologically distinct languages (Yadav et al., 2020). For example, if the Subject-Verb-Object (SVO) order is frequent in a language, the sentence processing should become easier when the order is preserved. Accordingly, since Italian is an SVO language, word order variation involving the relative ordering of subjects and objects may yield marked and more difficult to process sentences. This trend is particularly evident in the *Teacher* simplified sentences where the distribution of subjects preceding the verb is higher and the amount of direct objects preceding the verb is lower. We chose to exemplify this phenomenon by showing the following pair of sentences since it represents a quite typical example of how syntactic modifications may be strictly connected with what has been observed at the morpho-syntactic level of analysis:

O: E' un gran miracolo che io non abbia rinunciato a tutte le mie speranze perché esse sembrano assurde. **Le** conservo ancora, nonostante tutto, perché continuo a credere nell'intima bontà dell'uomo. [lit. It is a great miracle that I have not given up all my hopes because they seem absurd. **Them** I still keep, in spite of everything, because I continue to believe in the intimate goodness of man.]
S: Ma io ho fiducia, ho ancora *speranza* perchè credo nella bontà dell'uomo. [lit. But I have faith, I still have *hope* because I believe in the goodness of man.]

The two original sentences were merged into a unique sentence where the clitic pronoun *le* (“them”), operating as a direct object and, according to the Italian grammatical rules, preceding the main verb *conservo* (“keep”), was deleted. This yields a more simple sentence characterized by a canonical order of the verb's core arguments, where the referent of the pronoun, *speranza* (“hope”), serves as a direct object and follows the corresponding verb.

We think that the main results of this detailed analysis are two-fold. On the one hand, they show that the type of approach to construct a text simplification resource has an impact on the amount and strength of linguistic phenomena characterizing the original and simple corpora. Namely, the automatic approach yields a wider range of variations between the two versions, while the manual approach allows generating simple sentences that undergo stronger changes in terms of specific linguistic features. On the other hand, the analysis also highlighted several differences between the structural and intuitive manual approaches, which make the structural one more similar to the automatic one. This is the case for example of the simple lexicon, which increases more in the simple version of Terence and PaCCSS-IT than in *Teacher*, or of specific features related to the verbal morphology, which undergo more changes in the *Teacher*.

## 5. Conclusion

Text simplification is a topic that has received considerable attention in recent years in the computational linguistics community where it is more and more approached as a monolingual machine translation task. In this respect, the availability of large monolingual parallel data is a fundamental requirement to develop systems able to automatically infer the type of transformations that should be applied to “translate” the complex source text into the simple target text. In this article we addressed this topic from a quite less investigated point of view, i.e., the approach adopted in the construction of resources used for Automatic Text Simplification. We identified two main categories of resources, i.e., those simplified by human experts and those obtained through (semi)automatic approaches. We first surveyed existing ATS corpora for multiple languages from this perspective and we then focused on the Italian language for an empirical investigation based on available corpora. This choice was motivated by the fact that this is the only language, among less-resourced ones, for which not only there are resources representative of the “manual” and the “(semi)automatic” approach, but they are also large enough to allow a significant comparison. Comparing three different TS corpora aligned at the sentence level, we thus carried out a deep linguistic analysis of the main sentence phenomena characterizing the original and simple versions to investigate whether and to what extent the approach adopted for their construction can affect the internal composition of the resulting resources both in terms of undergone sentence transformations and linguistic properties.

From the point of view of the distribution of the main sentence transformations detected across paired corpora, we observed that some tendencies are shared in the simplification process, such as the deletion of redundant elements and the insertion of either words or phrases that make explicit missing or implicit information of a sentence. The perspective of linguistic profiling focused on the examination of the distribution of a wide variety of lexical, morpho-syntactic, and syntactic properties of the sentence has allowed us to better characterize the differences and similarities between automatically and manually derived corpora. In particular, we observe that a human-based simplification affects a relatively less number of linguistic features, yet the values of these features change more from the original to the simplified version. This is particularly the case of the “intuitive” approach, represented in our study by a corpus of texts simplified by teachers according to their feeling of the students' needs and without following predefined simplification rubrics. Instead, the automatically-derived corpus affects the whole structure of the sentence, though it yields less prominent differences for each feature.

Based on the results of both analyses we can also conclude that the automatic approach can deliver resources that not only are large enough to train an ATS system but, more importantly, that exhibit effective transformations toward the use of a simpler language. We believe that this is an important outcome especially if we consider that the method underlying the construction of PaCCSS-IT is language-agnostic and potentially transferable to other languages lacking available resources. However, we are also aware that an analysis of the internal linguistic composition of ATS resources is not sufficient to guarantee their quality. In this sense, a highly important aspect is represented by the human evaluation aimed at testing the effect of simplification in terms of text comprehension. This is also emphasized by the recent efforts of the ATS community toward the development of new metrics, such as SARI (Xu et al., 2016) and ASSET (Alva-Manchego et al., 2020a), which have become new standards for evaluating the quality of automatically simplified texts in light of their high correlation with human judgements of simplicity gain. In this respect, one possible way to expand this study could be to evaluate a subset of the corpus here considered concerning human judgments: this would allow us to assess if the current method of ranking sentence pairs for complexity is in line with the perception of sentence complexity by readers. Moreover, the multilingual perspective on linguistic profiling offered by a tool like Profiling-UD would provide a way to compare the distribution of the same linguistic phenomena within parallel corpora not only derived through different approaches but also across languages.

## Data Availability Statement

Publicly available datasets were analyzed in this study. This data can be found at: http://www.italianlp.it/resources/.

## Author Contributions

All authors listed have made a substantial, direct, and intellectual contribution to the work and approved it for publication.

## Conflict of Interest

The authors declare that the research was conducted in the absence of any commercial or financial relationships that could be construed as a potential conflict of interest.

## Publisher's Note

All claims expressed in this article are solely those of the authors and do not necessarily represent those of their affiliated organizations, or those of the publisher, the editors and the reviewers. Any product that may be evaluated in this article, or claim that may be made by its manufacturer, is not guaranteed or endorsed by the publisher.

## Footnotes

^1^ https://simple.wikipedia.org/wiki/Main_Page

^2^ https://cordis.europa.eu/project/id/257410

^3^ http://fr.vikidia.org

^4^See Paetzold and Specia (2017) for an up–to–date survey of methods and resources for lexical simplification.

^5^See, for instance, the Wikipedia guidelines for writing articles in Simple English. https://simple.wikipedia.org/wiki/Wikipedia:How_to_write_Simple_English_page.

^6^ www.universaldependencies.org

^7^Note that for the specific purpose of this study we considered both the UPOS, i.e., the set of Parts-Of-Speech defined by the UD project, and the XPOS, i.e., the set of POS specific for the Italian language that provides a finer-grained morpho-syntactic categorization of tokens.

^8^ https://universaldependencies.org/u/overview/complex-syntax.html#subordination
